# Elevated Plasma Reelin Levels in Children With Autism

**DOI:** 10.3389/fpsyt.2020.00242

**Published:** 2020-03-30

**Authors:** Inmaculada Cuchillo-Ibáñez, Patricia Andreo-Lillo, Lorena Pastor-Ferrándiz, Francisco Carratalá-Marco, Javier Sáez-Valero

**Affiliations:** ^1^Instituto de Neurociencias de Alicante, Department of Molecular Neurobiology and Neuropathology, Universidad Miguel Hernández-CSIC, Sant Joan d’Alacant, Spain; ^2^Centro de Investigación Biomédica en Red sobre Enfermedades Neurodegenerativas (CIBERNED), Sant Joan d’Alacant, Spain; ^3^Neuropediatric Unit, Pediatric Department, University Hospital of Sant Joan d’Alacant, Sant Joan d’Alacant, Spain

**Keywords:** reelin, autism, children, enzyme-linked immunosorbent assay, Western blotting, dimers, oligomers

## Abstract

Autism spectrum disorder (ASD) is a group of neurodevelopmental disorders involving age-dependent gene dysregulation. Reelin is a glycoprotein that varies its expression throughout lifetime and controls cortical patterning and synaptogenesis. Brain and plasma reelin levels have been reported to be low in adults with autism; as well as in children with autism, but only when compared to control adults. Therefore, reelin expression levels in children with autism are unclear. For this reason, we compared plasma reelin levels in children with autism and children without autism (non-ASD) of similar ages to evaluate reelin expression in ASD during childhood. Plasma samples from 19 non-ASD (8.9 ± 0.8 years) and 40 children with autism (7.5 ± 0.5 years) were analyzed. We found that 50% of the children with autism displayed similar plasma reelin levels to the non-ASD group. However, the remaining 50% expressed more than 30 times more reelin compared to non-ASD levels. We also show that male children with autism displayed significantly higher reelin levels than females. The clinical presentation of this subgroup could not be distinguished from that of children with autism. Epilepsy or attention-deficit/hyperactivity disorder (ADHD) was not associated to reelin levels. We conclude that the high levels of plasma reelin might be an important hallmark in a subset of children with autism, previously unnoticed. As we could not find any correlation between reelin levels and ASD clinical presentations, our results may indicate transient reelin increases in the plasma or the characterization of a group of ASD individuals with a different pathophysiology.

## Introduction

Autism spectrum disorder (ASD) is a heterogeneous group of neurodevelopmental disorders, characterized by deficits in social interaction and communication, as well as a wide range of stereotyped and repetitive behaviors. These characteristics are the basis of ASD diagnosis through behavioral tests and observation of the patients phenotype (1). In Europe, the average age of diagnosis of ASD is 3.5 years old, with great variability between countries. This is a fairly late age, given that some symptoms of ASD appear in the first year of lifetime, and highlights the gap between diagnosis and possible treatments (1).

A high proportion of the genes involved in ASD encode synaptic proteins, which has led to suggestions that ASD is a disorder of synaptogenesis (2, 3). One of these genes is *RELN*, which encodes for reelin protein. The Autism Sequencing Consortium in De Rubeis et al. (3) identified *RELN* with a 95% probability of being a gene whose anomalies directly contribute to autism. Reelin is a secreted signaling glycoprotein that regulates dendritic, axonal, and synaptogenesis development (4). During brain development, reelin controls neuronal migration and correct lamination in the neocortex, hippocampus, cerebellum, and spinal cord (5). Reelin function in non-neural tissues is not completely understood, however it is found in plasma. The major sources of reelin in the plasma are presumed to be the liver, platelets, and other differentiated blood cells (6). Although the regulation of gene expression is specific to each tissue, a parallelism has been observed between the mechanisms of epigenetic regulation in lymphocytes and the brain, including changes in methylation of the promoter of genes specifically related to synaptic function (7).

In the unique study where reelin levels have been quantified in plasma from individuals with autism (**Table 1**), reelin levels were reported to be lower in ASD children with respect to those in control adults, whereas no differences were found after comparison with siblings of similar age (8). Therefore, since ASD is a developmental disorder, it is important to examine reelin levels in children of related age, to assess the trend of potential changes in this protein before adulthood.

**Table 1:** Reelin expression in ASD.

Subjects	Average age (years) and n	Reelin expression changes in ASD with respect to Control		Reference
		Blood	Brain tissue	
Control adults *versus* children	Control adults = 38 ± 15 y; n = 8 ASD children = 7 ± 2 y; n = 13 twin pairs	Decrease		Fatemi et al., (8)
Children	Control siblings= 7 ± 3 y; n = 6 ASD children = 7 ± 2 y; n = 13 twin pairs	No changes		
Adults	Control adults = 23 ± 4 y; n = 8 ASD adults = 25 ± 5 y; n = 5		Decrease (only the 180-kDa fragment) in cerebellum	Fatemi et al., (9)
Adults	Control adults = 26 y; n = 10 ASD adults = 26 y; n = 7		Decrease (protein and mRNA) in frontal cortex and cerebellum	Fatemi et al., (10)
Adults	Control adults = 15-56 y; n = 11 ASD adults = 15-56 y; n = 6		Decrease (mRNA) in prefrontal cortex	Chow et al., (11)
Adults	Control adults = 34 y; n = 10 ASD adults = 30 y; n = 10		Increased binding of MeCP2 to *RELN* promoter in cerebellum	Zhubi et al., (12)
Adults	Control adults = 22 y; n = 10 ASD adults = 21 y; n = 10		Decrease (mRNA) in temporal cortex	Lintas et al., (13)

The table shows those reports where changes in reelin protein levels or reelin mRNA expression have been described in individuals with autism with respect to non-ASD subjects in different tissues.

ASD, autism spectrum disorder.

## Materials and Methods

### Patients and Interventions

The control/case study was designed with over 59 consecutive patients of Caucasian ethnic background (**Table 1**), coming from the neuropediatric outpatients’ clinic of a departmental university hospital, and all evaluated by the same neuro-pediatric team (Neuropediatric Unit, University Hospital of Sant Joan d’Alacant, Pediatric Department, Spain). All children passed through standard pediatric and neurological exams and needed routine blood workups. From them, 40 patients met the criteria for different ASD levels, following Diagnostic and Statistical Manual of Mental Disorders, Fifth Edition (DSM-V) criteria (14). The diagnosis was confirmed by a multidisciplinary team (pediatric neurologist, child psychiatrist, neuropsychologist, and social worker), and only in borderline cases, Autism Diagnostic Observation Schedule (ADOS evaluation) was used to confirm the diagnosis. The other 19 subjects did not meet any of the ASD criteria (named as non-ASD), but were attended by other minor neuropediatric circumstances such as the ones described below:

- Lobar benign infancy epilepsy (blood sample to test biochemical parameters)
- Single febrile seizures (blood sample to test infectious disease)
- Borderline or mild mental retardation (blood sample to test nutritional status).
- Migraine (blood sample to test iron status)
- Viral infections (blood sample to confirm biological markers in blood)

Blood extractions were always performed in the morning, around 9:00 am. The samples were collected in EDTA-containing sterile tubes and centrifuged (1800×rpm, 10 min) generally in less than 30 min after extraction. Plasma aliquots were stored at −80°C until use. Anonymous handling of the samples by the basic research unit was applied. This study was carried out in accordance with the recommendations of the “Ethics Committee” of the University Miguel Hernández de Elche and approved (reference IN.ICI.01.17). All subjects gave written informed consent in accordance with the Declaration of Helsinki.

### Measurement of Reelin by Enzyme-Linked Immunosorbent Assay

Commercial enzyme-linked immunosorbent assay (ELISA) kits (SEC775Hu, Cusabio Technology Llc) were used to quantify reelin in human plasma (50 µl of each plasma sample per well), according to manufacturer´s instructions.

### SDS-PAGE and Western Blotting

Plasma aliquots (1.5 µl) were boiled at 98°C for 3 min in 6× Laemmli sample buffer. All samples were assayed in at least two independent assays. After SDS-PAGE, the proteins were transferred to 0.45 µm pore PVDF membranes and detected with antibodies against reelin (N-terminal clone142, 1:400, Merck Millipore, Billerica, MA, USA) and transferrin (1:3000, Abcam, Cambridge, UK), which was used as a loading control. Primary antibody binding was visualized with fluorescently (IRDye) labeled secondary antibodies (1: 10000) and images were acquired using an Odyssey CLx Infrared Imaging system (LI-COR Biosciences GmbH).

### Statistical Analysis

The distribution of data was tested for normality using a D’Agostino-Pearson test. Data were analyzed and compared by groups, gender, and comorbid conditions using unpaired Student´s *t* test or Mann–Whitney test. For the correlation between age and reelin values, the “Spearman r” was calculated. The results are presented as the means ± SEM and all the analyses were performed using GraphPad Prism (Version 7; GraphPad Software, Inc). *p* value < 0.05 was considered significant.

## Results

**Figure 1:**
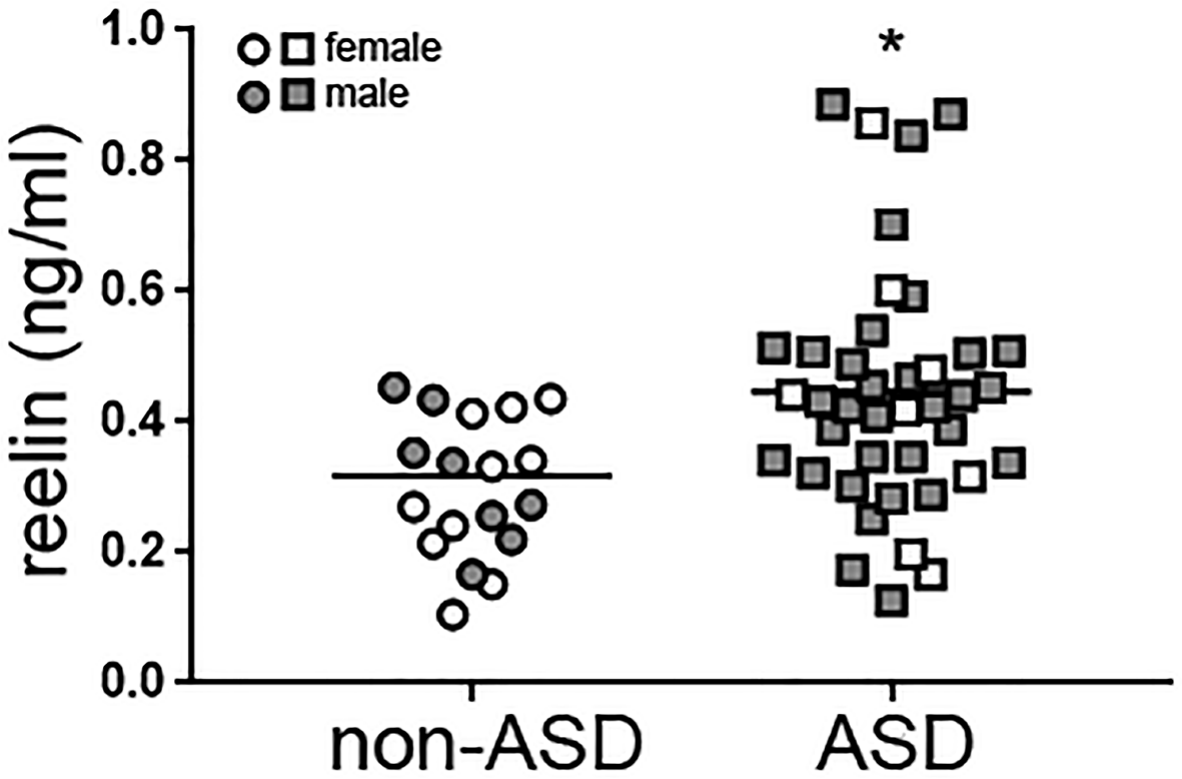
High reelin plasma levels in children with autism. Reelin from plasma samples from age-related children with autism (n = 40) and non-ASD (n = 19) was measured with a specific ELISA. Gender of the children is indicated. Mann Whitney test, *p = 0.0056. ASD, autism spectrum disorder; ELISA, enzyme-linked immunosorbent assay.

Reelin protein levels in plasma samples from children with autism and non-ASD age-related children (**Table 2**) were analyzed by ELISA assays (**Figure 1**). Reelin levels were higher in children with autism (0.44 ± 0.03 ng/ml, n = 40) with respect to those in non-ASD children (0.32 ± 0.03 ng/ml; n = 19; *p* < 0.01) When comparing by gender, no differences were found between male and female children in overall. However, reelin levels in males with autism were significantly higher (0.44 ± 0.03 ng/ml) than those in the male non-ASD group (0.30 ± 0.03 ng/ml; *p* = 0.01), while no differences were found between female children. No correlation was found between age and reelin levels, although we observed a tendency of a positive correlation between age and male children with autism (Pearson *r* = 0.31, *p* = 0.053). Stratification by epilepsy or attention-deficit/hyperactivity disorder (ADHD) (attention deficit hyperactivity disorder) did not show statistical differences in reelin levels when all children were analyzed.

**Table 2:** Distribution by age and gender of children with autism and non-ASD children of this study.

	Gender	n	Age (y)	Age range (y)	Age median (y)	Epilepsy (n)	ADHD (n)
Non-ASD	Male	9 (47%)	9.40 ± 1.17	1.99–13.56	9.87	4	1
	Female	10 (53%)	8.42 ± 1.29	2.43–14.69	9.76	3	0
	All	19	8.88 ± 0.86	1.99–14.69	10.31	7 (37%)	1 (5.2%)
ASD	Male	32 (80%)	7.42 ± 0.61	1.9–14.28	7.65	10	8
	Female	8 (20%)	7.64 ± 1.46	2.78–14.52	7.94	7	1
	All	40	7.47 ± 0.56	1.90–14.52	7.65	17 (42.5%)	9 (22.5%)

Age is expressed as mean [in years (y) ± SEM]. Cases with comorbid situations such as epilepsy and/or ADHD are displayed. Percentages are expressed respect to “All” in each group.

**Figure 2:**
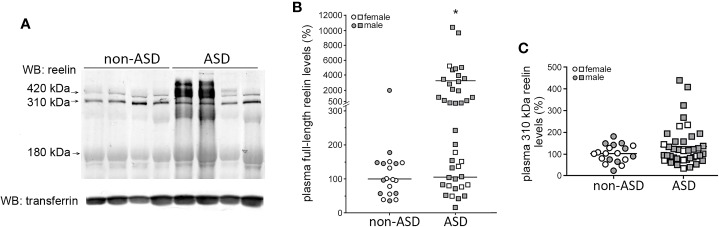
Two subgroups among the ASD subjects are defined with respect to plasma full-length reelin levels. **(A)** Representative Western blot of plasma reelin and transferrin (loading control) from children with autism and non-ASD children. Arrows indicate the full-length protein (420 kDa), the 310-kDa N-terminal fragment, and the faint 180-kDa fragment. **(B)** Quantification of full-length reelin intensity normalized with respect to transferrin, represented using a broken y-axis due to a large data spread (n = 40 children with autism, n = 19 non-ASD children). Mann–Whitney test, *p < 0.0001, “high ASD reelin” *vs* “normal ASD reelin” and “high ASD reelin” *vs* “non-ASD reelin.”**(C)** Quantification of the 310-kDa reelin fragment intensity, normalized respect to transferrin (n = 40 children with autism, n = 19 non-ASD children). Gender of the children is indicated. Mean of the values are represented as horizontal lines.

We further analyzed plasma reelin levels by Western blot, which allowed the quantification of the full-length reelin (420 kDa) and separately, the N-terminal proteolytic fragments (**Figure 2A**). However, among these fragments, only the major 310-kDa fragment was clearly detected, not the 180-kDa fragment. Full-length reelin levels were significantly higher in children with autism (~17 times) with respect to non-ASD children (*p* = 0.0028). When these reelin values were plotted, a wide spread of reelin levels was observed in the ASD group. For the analysis, a histogram of the frequency distribution of ASD values was performed and two subgroups were distinguished (**Figure 2B**). The first ASD subgroup (n = 20) displayed full-length reelin levels which were not significantly different (105% ± 13%, range 16%–240%) to those found in the non-ASD group after normalization (100% ± 10%, range 36%–178%). A second ASD subgroup (n = 20) did not follow a normal distribution and displayed higher full-length reelin levels than both the non-ASD group and the first ASD subgroup (3275% ± 644%, range 290%–10400%, *p* < 0.0001). In the non-ASD group, only 1 out of 19 children displayed high reelin levels of ~20 times higher than the remaining cases. The 310-kDa reelin fragment levels did not show significant differences between groups (**Figure 2C**).

Comparison of full-length reelin levels by gender revealed higher levels in male children with autism with respect to females with autism (~300% increment, *p* = 0.014), which is consistent with the distribution of high full-length reelin levels by gender (non-ASD cases: 1 out of 9 male children, no female children with high reelin levels; ASD cases: 18 out of 33 male children, 1 out of 7 female children with high reelin levels). Correlation of full-length reelin levels with age was not found, neither was any association with epilepsy or ADHD.

## Discussion

We have found that ~50% of the analyzed children with autism expressed extremely high levels of reelin in blood (according to Western blotting assays). The stratification of ASD cases according to plasma reelin levels was independent of age or any other comorbid conditions such as epilepsy, despite some previous reports having found a correlation between this disorder and reelin (15, 16). The only study so far reporting measurements of reelin in plasma in ASD (8) showed differences in reelin levels in children with autism as compared to control adults, but not to their phenotypically normal age-matched siblings and their parents, suggesting a familial variability of reelin levels that is not associated with ASD. Furthermore, although they also employed Western blot analysis to measure reelin levels, we believe the underlying reason behind this discrepancy might be a degradation of reelin in their samples. Their blots show the full-length reelin protein and two proteolytic fragments (310 and 180 kDa). In our experience, the presence of abundant levels of the 180-kDa fragment may be a sign of reelin degradation in blood; whereas in brain extracts this fragment is abundant (17). Reelin in human plasma is sensitive to proteolysis, freeze-thawing and heating during long-term storage (18, 19). Another complementary explanation is that the notable differences we observed were only seen in ~50% of the cases, representing an undefined subset of children which might have been under-represented in the previous study (8). A smaller sample size in the previous studies in this area (**Table 1**) might therefore explain why the subjects with high reelin levels were previously not observed.

In our study, male children with autism displayed higher levels of reelin than females with autism, analyzed by ELISA and by Western blot. Little is known about the gender dimorphic expression of reelin, but some mice models show gender-associated reelin expression and more vulnerability to reelin expression changes in males (20, 21). Specific hormones could be candidates as regulators of reelin expression, such as the sex hormones testosterone and estrogen, which have been reported to modulate expression of *RELN* (22, 23). The neuropeptides oxytocin and vasopressin, whose effects on brain and behavior are sexually dimorphic, especially during the course of development, have been implicated in autism and are likely involved in reelin expression regulation (23).

Whole-genome analysis of cortex mRNA levels has revealed age-dependent dysregulation of specific pathways. In children with autism (2–14 years), genetic pathways regulating cortical patterning, cell number and differentiation are dysregulated, whereas in adults with autism (15–56 years), dysregulation affects signalling, and repair pathways (11). In adults with autism, reelin expression is lower in prefrontal cortex, frontal cortex, temporal cortex, and cerebellum [(11, 15, 17, 18), **Table 1**]. Thus, it would be important to determine if these increments in plasma reelin levels represent a transient phenomenon from childhood to adulthood that could be controlled by reversible mechanisms, such as epigenetic regulation. Accordingly, the epigenetic regulation of *RELN* varies throughout development, in particular, methylation of *RELN* promoter in the temporal cortex shows little methylation before puberty (allowing protein expression), in contrast to a strong post-pubertal methylation which reduces expression of reelin (12, 13). Furthermore, methylation of the *RELN* promoter in the peripheral blood was reported to be different in male and female individuals (24). In our study, reelin levels only from male children with autism tended to increase with age, suggesting the possibility of a different epigenetic mechanism with respect to female children with autism, and likely with respect to non-ASD children as well. Overall, our data strongly suggests a gender dimorphic expression of reelin in ASD, where increased reelin levels in males would be associated with ASD. Further studies including larger number of subjects are required to confirm this gender-associated trend.

Single nucleotide polymorphisms (SNPs) in *RELN* have been significantly associated with an increased risk of ASD (25) as well as *de novo* mutations in *RELN* with a high probability of being pathological (26, 27). It would be interesting to determine whether the children with autism and high reelin levels of this study carry *RELN* variants that affect its expression, and to test whether these children express other variants affecting genes in the reelin pathway.

We found greater differences between non-ASD children and children with autism and when reelin expression levels, from the same samples, were measured by Western blot than when they were evaluated by ELISA. While in the denaturing-conditions of Western blot reelin monomers were analyzed, the ELISA assay was likely measuring native reelin homodimers, the active form of reelin that binds to the receptors (28). Therefore, the fact that children with autism display a large increase in reelin monomers levels after denaturation suggests the existence of a higher proportion of reelin dimers/oligomers in the plasma of these children with regards to non-ASD children. Differences in reelin complexes have been reported in other neurological condition such as Alzheimer’s disease (29, 30). Remarkably, recent data indicate that commercial anti-reelin antibodies display different reactivity to monomeric or dimeric reelin protein (31), which highlights the relevance of an appropriate methodological design for reelin quantitative analyses.

Our results raise new questions about the physiological relevance of high reelin expression in ASD at early lifetime. It would be important to confirm whether children with high reelin levels develop different ASD clinical presentations later on, since reelin function is related to migration and synaptogenesis development and such mechanisms have been found to be dysregulated in ASD (32, 33). This scenario might indicate that altered reelin plasma levels could contribute to the progression and severity of the disease during childhood.

Children in this study were younger than 15 years old and belonged to a “compulsory referral population” of 33,731 children. This population includes all the children belonging to our regional health department and also covers refugees or migrants, who are registered in the database with free health assistance. We therefore believe our results to be of epidemiological relevance as our dataset represents 1.07% of the estimated 363 autistic children in our regional catchment area. Nevertheless, given the preliminary nature of this study, our next aim will focus on replicating this study in other cohorts as well as in the comparison to a group without neurological disorders, as typically developing children.

## Data Availability Statement

The datasets for this article are not publicly available because participants recruited in this study did not give their consent to their raw data being publicly shared even if anonymised. Additional information regarding the datasets should be directed to icuchillo@umh.es.

## Ethics Statement

The studies involving human participants were reviewed and approved by Ethic Committee from "Órgano Evaluador de Proyectos" of "Vicerrectorado de Investigación e Innovación" of "Universidad Miguel Hernández de Elche". Written informed consent to participate in this study was provided by the participants' legal guardian/next of kin, in accordance with the Declaration of Helsinki.

## Author CONTRIBUTIONS

IC-I, JS-V and FC-M designed the study, performed the experiments and the analysis and wrote the manuscript. PA-L, LP-F and FC-M evaluated the children and performed the pediatric and neurological exams.

## Funding

This work was supported by grants from Fondo de Investigaciones Sanitarias (PI15/00665 and PI19-01359, co-funded by the Fondo Europeo de Desarrollo Regional, FEDER "Investing in your future"), by the Direcció General d’Universitat, Investigació i Ciència, GVA (AICO/2018/090) and through CIBERNED (Instituto de Salud Carlos III, Spain). We also acknowledge financial support from the Spanish Ministerio de Economía y Competitividad, through the “Severo Ochoa” Programme for Centres of Excellence in R&D (SEV-2017-0723).

## Conflict of Interest

The authors declare that the research was conducted in the absence of any commercial or financial relationships that could be construed as a potential conflict of interest.
